# Determinants of Zika Transmission and Control

**DOI:** 10.1093/infdis/jiy691

**Published:** 2018-12-20

**Authors:** Hannah Clapham

**Affiliations:** Oxford University Clinical Research Unit, Ho Chi Minh City, Vietnam

**Keywords:** Zika, vaccine development, transmission route, epidemiology, health economics

**(See the Major Article by Bartsch et al, on pages 920–31; Rosenberg et al, on pages 932–40.)**

This issue of *The Journal of Infectious Diseases* contains 2 articles focusing on different aspects of Zika virus (ZIKV) transmission and control. Until 2015, Zika was little known, and little was known about ZIKV. With the 2015–2016 epidemics leading to large numbers of cases in multiple countries [1], and with the realization that ZIKV was causing congenital defects, understanding and mitigating virus transmission became a global health priority [2]. However, in the last 2 years, there has been much less ZIKV transmission identified, and Zika has slipped somewhat from public view. The World Health Organization (WHO), however, remains committed to a long-term response [2], and much research continues to be published.

The most worrying aspect of ZIKV is the ability to cause congenital Zika syndrome. The impact of this from the 2015–2016 epidemics will continue to be felt for many years and continued research support is necessary to understand it [3]. Guillain-Barré syndrome is also a severe outcome of ZIKV infection, though with some uncertainty about the rate at which it occurs [4]. One of the most fascinating unknowns, also important for control, concerns the role of the 2 major routes of transmission: vector-borne and sexual. For understanding sexual transmission, many investigators have focused on the longevity of virus persistence in different bodily fluids, and what that could mean for transmission (summarized in [5]).

Because of the seemingly low levels of ongoing ZIKV transmission, studies from the 2015–2016 epidemics are the most important current resource for understanding the drivers of virus transmission. In one important article in this issue of *The Journal of Infectious Diseases*, Rosenberg et al report on a study in Puerto Rico regarding virus transmission in households of individuals infected with ZIKV. The focus on households within areas of ongoing transmission provides the possibility to disentangle the roles of the 2 major routes of transmission. The results were mixed, depending on how Zika was diagnosed and what pairs were considered. Looking at all pairs, the authors found that pairs with sexual contact were 2.2 times more likely (95% confidence interval [CI], 1.1–4.5) to both be polymerase chain reaction positive than other pairs, but there was no difference regarding immunoglobulin M positivity. This result adds to the evidence to be used for developing guidance given to infected individuals about protecting others from infection. This information is particularly useful coming from an area of ongoing mosquito transmission, as previous studies highlighting the role of sexual transmission have mainly come from travelers returning to areas without mosquito transmission [6, 7]. However, as the authors note, though for a given pair the risk of sexual transmission may be twice that of via a mosquito bite, on a population level the proportion of transmissions due to sexual transmission is low.

As well as quantifying sexual vs mosquito transmission, this study also showed that household contacts were more likely to be infected if their homes had open and unscreened doors and windows (2.5 times [95% CI, 1.5–4.1] as likely) or had open windows and doors with screens (2.1 times [95% CI, 1.2–3.6]). This highlights that basic ways of protecting an individual against ZIKV transmission can reduce risk. Therefore, individuals and households should continue to be advised and supported to put in place these relevant protections.

There are some limitations to the Rosenberg et al study that should stimulate future research questions. Though the study did have some prospective follow-up of cases, this was at 2–4 months, and perhaps this was the reason that few incident infections were found. As the authors note, the sexual risk is confounded by the fact that pairs with a sexual relationship are probably also more likely to have other different types of contacts. Future research in households, including collecting more detailed information about types of contacts, could help disentangle this further. Finally, as the authors note, further studies are needed on the relative risks of male-to-male, female-to-male, female-to-female, and male-to-female sexual transmission.

Though important, protecting against mosquito bites and sexual transmission would appear to be insufficient to completely control *Aedes aegypti*–borne virus transmission. Therefore, any available vaccine against ZIKV would be an important part of effective future control. At the time of high ZIKV transmission, there were reports of 18 groups working on ZIKV vaccine, some with multiple vaccines at some stage of development; however, development of many of these vaccines appears to have been halted, with WHO currently listing 8 in development [8]. Much vaccine development stalled because as transmission waned, the global interest also waned and, therefore, the economic incentive for developing a vaccine may be less strong. To assess whether this is the case, the second Zika study reported in this issue of *The Journal of Infectious Diseases*, by Bartsch et al, focuses on determining the value of various ZIKV vaccination strategies.

Bartsch et al considered the costs and impact of different ZIKV vaccination strategies in 3 countries: Honduras, Brazil, and Puerto Rico. The authors used a transmission model for ZIKV transmission and an economic model to consider the costs of disease and vaccination. The study considered strategies of vaccinating everyone, or targeting school-aged children, young adults, or women of childbearing age.

The authors performed rigorous sensitivity analyses concerning vaccine cost, efficacy, coverage and targeting of vaccine campaigns, and transmission intensity and how soon after vaccination the outbreak occurred. They estimated that ZIKV vaccination would be cost-effective under some of the scenarios considered, and the details of such work will be important for developing relevant vaccine target profiles. Vaccination was more often cost-saving or cost-effective in Brazil and Puerto Rico than in Honduras, except at low cost and/or high transmission rates. The authors found that targeting young adults or women of childbearing age was the most cost-effective strategy. This result is presumably due to the more direct effect of these strategies on, and the high costs of, congenital Zika syndrome. For the same reasons, these targeting strategies were particularly more effective compared to targeting school-aged children if the outbreak occurred sooner after vaccination started. This result, however, may be sensitive to not just the overall vaccine efficacy, but the specific profile of any vaccine with respect to protection against congenital Zika syndrome.

Importantly, the main scenario considered is one in which the outbreak does not happen until 5 years after vaccination started. This is one of the major concerns with continued development of a ZIKV vaccine: that money would be spent on vaccination, but that little to no transmission would have happened anyway. Indeed, the future trajectory of ZIKV transmission is very uncertain. Modeling simulations have predicted that in areas of high transmission in 2015–2016, there will be low transmission until at least 2018 [9] or for up to 10 years [10], until population susceptibility to ZIKV has been reestablished, though with smaller outbreaks possible before that. It is reassuring that in the Bartsch et al study, even if an outbreak occurred 5 years after vaccination started, vaccination was cost-effective under some scenarios. However, it would be interesting to assess cost-effectiveness if the next large outbreak of Zika did not happen for an even longer period of time. There are also a number of epidemiological uncertainties to be further considered to fully assess the global cost-effectiveness of ZIKV vaccination. In a similar study in Colombia, preexisting herd immunity was seen to be an important determinant of cost-effectiveness [11]. Preexisting immunity is particularly important in determining the future population risk of congenital Zika syndrome, a seemingly large driver of the costs, as this risk is presumably determined by the immunity to ZIKV infection in women of childbearing age. The model also did not include ZIKV sexual transmission; perhaps the results of the Rosenberg et al article could help inform this in future work.

Zika is not gone forever. Indeed, there are low levels of ZIKV transmission ongoing in a number of countries [1, 12], and the future potential for ZIKV to have a large impact remains unclear. Mathematical modeling studies like the one reported here can help us understand the future risks, but to understand the epidemiology of Zika moving forward, we also need continued surveillance to identify areas with active virus transmission. This is necessary wherever ZIKV transmission occurs, and is currently limited by difficulties in ZIKV laboratory testing. As illustrated in the article by Rosenberg et al, different tests on different fluids are positive at different times. For successful surveillance, development of reliable diagnostics remains a priority [13], particularly in places with co-circulation of other flaviviruses. Even if an economic argument for vaccine development can be made, in times of low or uncertain transmission there remains the challenge of how to actually test vaccines, and perhaps human challenge models will be needed [14]. Despite all these uncertainties and difficulties, we know that ZIKV has had the ability to have a large detrimental health impact, particularly through congenital Zika syndrome. We must continue to collect the relevant information to help weigh the future risks from ZIKV and the need for ZIKV vaccine development.

## Notes

***Financial support.*** The author is funded by the Wellcome Trust (grant number 089276/B/09/7).

***Potential conflicts of interest.*** Author certifies no reported conflicts of interest. The author has submitted the ICMJE Form for Disclosure of Potential Conflicts of Interest. Conflicts that the editors consider relevant to the content of the manuscript have been disclosed.
